# Sexual Dimorphism in Fin Size and Shape in North American Killifish

**DOI:** 10.1002/ece3.71194

**Published:** 2025-04-11

**Authors:** Elijah J. Davis, Kasey Brockelsby, Milton Tan, Rebecca C. Fuller

**Affiliations:** ^1^ Department of Evolution, Ecology, and Behavior, School of Integrative Biology University of Illinois Urbana Illinois USA; ^2^ Prairie Research Institute, Illinois Natural History Survey University of Illinois Champaign Illinois USA

**Keywords:** Cyprinodontiformes, fin shape, median fins, sexual dimorphism, sexual selection

## Abstract

Sexual dimorphism is intriguing because it suggests that males and females differ in phenotypic optima for traits and that sex‐specific trait values can evolve despite a shared genome. Differences in sexual dimorphism across populations or species suggest that the nature of sexual selection and/or genetic constraints differ among species. Here, we measured sexual dimorphism in 20 species of North American killifish (Fundulidae) in size and shape of dorsal, anal, and caudal fins. We observed profound sexual dimorphism in anal and dorsal fin size and shape across all species, suggesting a common direction of selection. Sexual dimorphism was also present in caudal fin size and shape but was much lower in magnitude, with several species not differing from a null expectation of zero. There was little evidence for a phylogenetic signal in the levels of sexual dimorphism in dorsal and anal fin traits. We also found a strong phylogenetic correlation between sexual dimorphism in anal and dorsal fin shape but no phylogenetic correlation between fin area, base length, or ray length across different fins. Our results indicate that there is pronounced sexual dimorphism in anal and dorsal fin size and shape across fundulids. Similar patterns of sexual dimorphism in anal and dorsal fin properties have been documented in other groups, including gars, bichirs, graylings, minnows, and many species in the Atherinomorpha, suggesting that this pattern may be common across Actinopterygii.

## Graphical abstract

Sexual dimorphism is pervasive in North American killifish in the size and shape of their dorsal and anal fins. These fins perform multiple functions, including grasping females during spawning, dominance displays towards rival males, and courtship towards females. Sexual selection may share a common direction across this group. Photo Credits: (Top) Lance Merry, (Bottom) Elijah Davis.

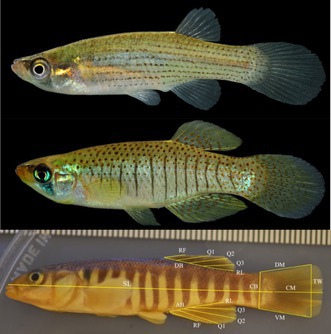

Sexual dimorphism, characterized by phenotypic differences between males and females, reflects the outcome of sex‐specific natural and sexual selection (Owens and Hartley 1998; Dawson and Gerber 1999). Sexually dimorphic traits are interesting for various reasons. Males and females share a common genome, raising questions as to how these traits are expressed, whether selection acting on one sex can induce correlated responses in the other, and to what extent these traits can evolve independently in each sex (Pomiankowski 1987; Williams and Carroll 2009). In comparative studies, sexual dimorphism is often used to infer the strength of sexual selection, with the inference being that high levels of sexual dimorphism indicate strong sexual selection (Kodric‐Brown 1985; Williams et al. 2013; Delhey et al. 2023).

Many teleost fishes possess sexual differences in a variety of traits, including coloration, tubercles, territoriality, parental care, and fin morphology (Mendoza 1965; Sargent and Gross 1993; Ah‐King et al. 2005; McMillan et al. 2013; Kottler and Schartl 2018). While differences in coloration, tubercles, and behavior are easily observable, variation in fin size and shape is often less apparent, especially in smaller fish. Nevertheless, the evolution of sexual dimorphism in fin traits is intriguing because fins serve multiple functions. In addition to their roles in swimming and maneuverability, fins are often involved in aggressive displays towards competing males, courtship displays towards females, and clasping females during spawning.

Sexual dimorphism in fin traits has been documented in multiple groups in the Cyprinodontiformes, particularly in the Poeciliidae. Studies of swordtail fishes have sought to determine the evolutionary origins of the caudal sword in males and whether this evolved concurrently or after the evolution of female mating preferences for the sword (Basolo 1990; Basolo 1995; Rosenthal and Evans 1998; Rosenthal et al. 2001; Rosenthal et al. 2002; Wong and Rosenthal 2006). Similar studies have been conducted in mollies focusing on the evolution of dorsal fin size and the associated female preference across short‐finned and large‐finned molly species (Kozak et al. 2008). These studies were concerned with the evolution of male traits and female mating preferences but did not explicitly consider the evolution of sexual dimorphism per se. More recently, a comparative study of sexual dimorphism in dorsal fin size in poeciliids showed that the appearance of sexual dimorphism was first associated with male competition for mates and then later for display traits to females (Goldberg et al. 2019). Sexual dimorphism in anal and dorsal fins has also been documented in one species of oriental killifish, *Aphaniops stoliczkanus* (family Aphaniidae) (Mainero et al. 2023).

Sexual dimorphism in dorsal and anal fin traits is also present in the medakas in the order Beloniformes, which is closely related to the Cyprinodontiformes in the superorder Atherinomorpha. A comparative study of medaka (*Oryzias* spp.) has shown dimorphism in body size and fin length, with varying levels of dimorphism between northern and southern populations (Sumarto et al. 2020). Morphological studies like these give insight into the traits that may undergo sexual selection and how environmental factors affect their evolution.

Our study focuses on fin size and shape evolution in the unpaired fins of North American killifish (Fundulidae). There are several compelling reasons to study sexual dimorphism in Fundulidae. First, many species descriptions indicate the presence of sexual dimorphism in fins, with males possessing large dorsal and anal fins (Arndt 1971). Such differences have been thoroughly documented in the blackstripe topminnow, *Fundulus notatus* (Welsh et al. 2013; Welsh and Fuller 2015). Species descriptions also suggest fin shape is sexually dimorphic and that this dimorphism is likely to differ across species, with topminnows appearing to have large dorsal and anal fins that are ‘pointy’ posteriorly. In contrast, other species have males with large, rounded dorsal and anal fins (Page and Burr 2011). Second, fundulids are also interesting because male nuptial colors often differ among species and even populations within species, which may be consistent with differing levels of sexual selection. Bluefin killifish, *Lucania goodei*, are exceedingly dimorphic in anal and dorsal fin coloration with pronounced variation within and among populations (Fuller and Travis 2004; Fuller et al. 2005; McGhee et al. 2007; Fuller and Noa 2010; Fuller et al. 2010; Johnson et al. 2018; Mitchem et al. 2018; Fuller et al. 2022). Schaefer and colleagues (2012) have similarly studied the evolution of sexually dimorphic spot patterns on the fins of male and female *Fundulus olivaceus* . Hence, sexual selection on fin traits appears to be common in this group. Third, fundulids span a wide range of habitat types, which may also affect the strength of sexual selection and the resulting dimorphism. As a family, they span a large latitudinal gradient that causes variation in the length of breeding and growing seasons (Foster 1967). Fundulids are also well‐known for their ability to tolerate dramatic swings in temperature, salinity, oxygen, and pollution (Dunson and Travis 1991; Fuller et al. 2007; Fuller 2008; Whitehead 2010; Berdan and Fuller 2012; Whitehead et al. 2012a; Whitehead et al. 2012b; Brennan et al. 2015; Brennan et al. 2016). These patterns suggest that fundulids are capable of rapid evolution as a function of both natural and sexual selection.

In this study, we quantified sexual dimorphism in dorsal, anal, and caudal fin traits across several fundulid species. We asked the following questions: Which fins show evidence of sexual dimorphism in size and shape? Are the levels of sexual dimorphism similar across Fundulidae, or are there significant differences among species? Similarly, is there a phylogenetic signal that would indicate that similarities between closely related species are due to evolutionary relatedness? Finally, are there strong correlations in sexual dimorphism across different fins that would indicate pleiotropy and shared developmental pathways during fin growth? To answer these questions, we photographed males and females from twenty species belonging to the Fundulidae family. We digitally measured the size and shape of the dorsal, anal, and caudal fins. While there were some minor differences in levels of sexual dimorphism across species, the overall picture was one of remarkably similar levels of dimorphism, with the dorsal and anal fins showing large differences in size and shape as a function of sex.

## 1 Materials and Methods

Our goal was to measure sexual dimorphism in the size and shape of the unpaired fins (dorsal, anal, and caudal fins) across Fundulidae. We measured 364 individuals across 20 species (*F. blairae, F. catenatus, F. chrysotus, F. diaphanus, F. dispar, F. grandis, F. heteroclitus, F. lineolatus, F. majalis, F. notatus, F. notti, F. olivaceus, F. pulvereus, F. rathbuni, F. seminolis, F. similis, F. zebrinus, Lucania goodei, L. parva, Leptolucania ommata*). We chose these species because they were present in sufficient numbers for both sexes in the collection at the Illinois Natural History Survey. Table 1 lists the species, sample sizes for each sex, lot numbers/identifiers, and the year each was collected. These samples were collected between 1938 and 2019. Samples were fixed in formalin and stored in 70% ethanol. Most of the samples were collected between 1960 and the late 1990's. Males and females were identified externally using known sexually dimorphic body pigmentation phenotypes that did not rely on fin traits (Page and Burr 2011). Sexually immature specimens and those whose sex could not be clearly determined were excluded. We also excluded preserved individuals whose fins could not be visualized (i.e., could not be pulled away from their bodies and unfolded). A minimum of six individuals were measured from each sex. For most species, 10 individuals were measured per sex.

**TABLE 1:** Species, sample sizes for males and females, and INHS (Illinois Natural History Survey) lot numbers (years) and other identifiers.

Species	Males	Females	Lot # (year) and identifiers
*Fundulus blairae*	6	9	41,186 (1997), 87,096 (1980), Bayon Boeuf Dec 1967, Lake of Pines March 1967
*Fundulus catenatus*	10	10	30,225 (1993), 30,259 (1993), 30,285 (1993), 79,281 (1978), 80,756 (1978), 81,695 (1978), 82,745 (1978), 82,769 (1978), 84,184 (1978), 87,329 (1981)
*Fundulus chrysotus*	10	10	87,108 (1980), 38,739 (1996), 41,187 (1997), Bayon Boeuf 1967, Canal 1 1/21966 Smith, Small Lake 1966 Smith, Hemp Creek 1967
*Fundulus diaphanus*	10	10	4162 (1964), 106,420 (1970), 106,939 (2013)
*Fundulus dispar*	10	10	4084 (1965), 15,066 (1967), 73,887 (1968), 79,785 (1969), 91,483 (2001), 98,660 (2003), 99,487 (2004), 110,059 (2015), 112,940 (2019)
*Fundulus grandis*	10	10	58,015 (1967), 67,349 (1951), 79,492 (1964), 87,032 (1980), 87,070 (1980)
*Fundulus heteroclitus*	10	10	51,727 (1970)
*Fundulus lineolatus*	10	10	38,165 (1996), 57,881 (1981), 64,714 (1989), 74,441 (1966), 87,527 (1981), 165,590 (1998)
*Fundulus majalis*	7	6	87,494 (1981), 87,535 (1981), 87,538 (1981), 165,529 (1954), 165,686 (1938)
*Fundulus notatus*	10	9	94,981 (1988), 95,482 (1987), 95,524 (1990)
*Fundulus notti*	10	10	57,951 (1974), 76,212 (1977)
*Funudulus olivaceus*	10	10	57,206 (2000), 88,801 (2000), 89,402 (2000), 91,927 (2001), 92,208 (2001), Blackman Creek 1959
*Fundulus pulvereus*	6	8	57,971 (1969), 79,493 (1964), 79,799 (1951), 87,031 (1980), 87,694 (1982), 164,614 (1959)
*Fundulus rathbuni*	10	10	75,077 (1976), 75,218 (1976), 75,255 (1976), 87,534 (1981), 88,240 (1983), 164,407 (1982)
*Fundulus seminolis*	10	7	164,512 (1991), 164,513 (1988), Lake Hamilton 1966
*Fundulus similis*	6	7	51,697 (1968), 83,131 (1967), 83,133 (1969), 164,443 (1978), Port Isabel 1975
*Fundulus zebrinus*	10	10	41,323 (1997), 83,283 (1969)
*Leptolucania ommata*	9	10	62,936 (1987), 64,725 (1989), 74,407 (1968), 74,689 (1963), 74,756 (1968), 164,735 (1994)
*Lucania goodei*	10	10	33,022 (1994), 63,745 (1987), 63,951 (1988), 64,726 (1989), 74,446 (1972), 74,709 (1974)
*Lucania parva*	10	10	81,970 (1965), 83,443 (1963), 87,765 (1982)

Each individual was photographed alongside a ruler using a Nikon D5600 with an attached AF‐S Micro Nikkor 105 mm lens. For each fish, we took two photos: one of the whole body and one zoomed in on the posterior of the fish with the anal, dorsal, and caudal fins included in the image. We chose to exclude the pectoral and pelvic fins. Photographing these fins would have required removing them from the body. In addition, an unpublished study from our lab indicates that the levels of sexual dimorphism are modest for pectoral and pelvic fins (Fuller and Brockelsby, unpublished manuscript).

**FIGURE 1:**
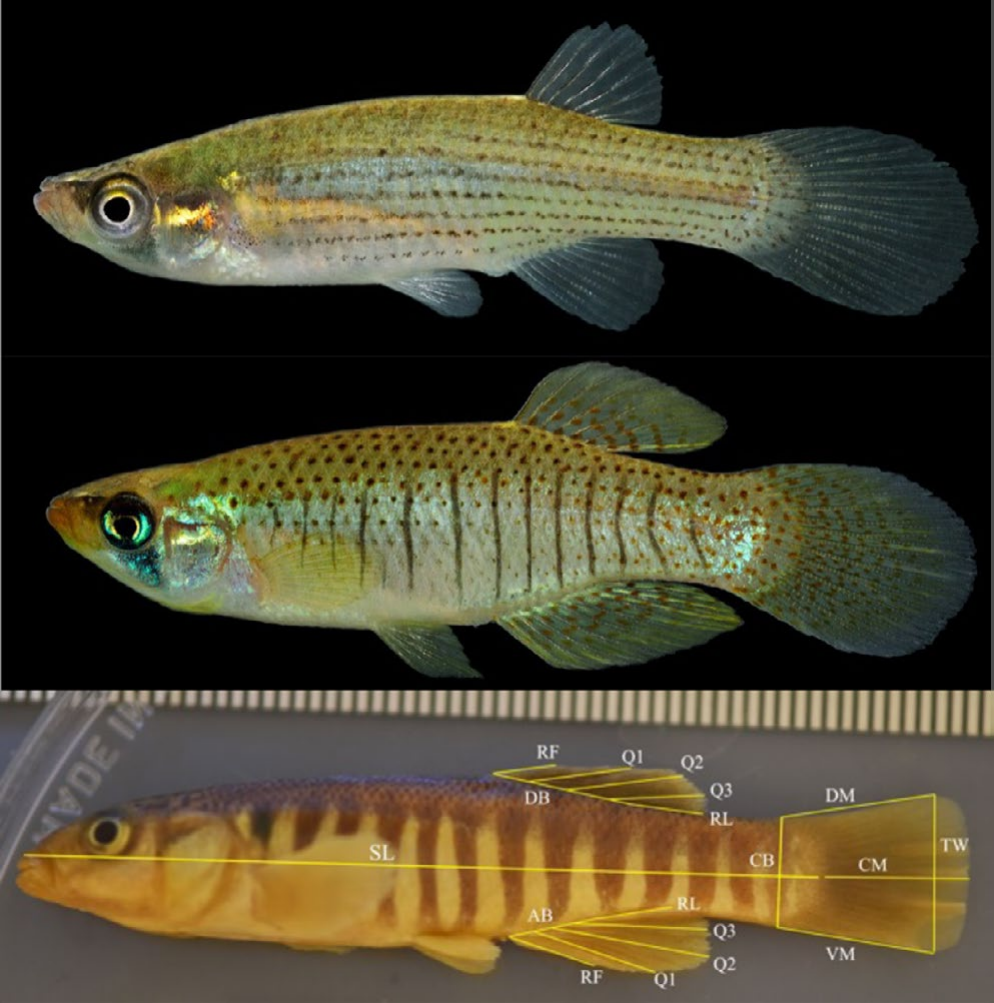
Top two images show female (top) and male (bottom) *F. dispar*. Photo credit: Lance Merry. Bottom image shows a preserved specimen of *F. zebrinus* . Photo credit: Elijah Davis. Standard length (SL), dorsal base length (DB), anal base length (AB), first fin ray length (RF), first quartile fin length (Q1), median quartile fin length (Q2), third quartile fin length (Q3), last fin ray length (RL), caudal peduncle length (CP), dorsal margin caudal fin length (DM), middle caudal fin length (CM), ventral margin caudal fin length (VM), caudal fin area (CA).

Photographs were analyzed using ImageJ to collect measurements on 18 morphological variables: standard length and unpaired fin measurements. Standard length was measured as the distance from the most anterior portion of the fish snout to the caudal peduncle. The dorsal and anal base measurements reflect the distance from the anterior and posterior fin insertion points. We measured fin ray lengths at five locations: the first and last fin ray, the median fin ray, and the two fin rays intermediate between the median and distal fin rays (Figure 1). We refer to these intermediate fin rays as the first and third quartile fin rays and the median fin ray. For the caudal fin, we measured the caudal peduncle width and three traits. The dorsal and ventral margins were measured as the distance from the caudal peduncle to the point where the fin curves. The middle fin measurement was measured from the caudal peduncle to the tip of the median fin ray.

For the dorsal and anal fin areas, we calculated areas as the average of the five fin ray measurements multiplied by the dorsal/anal base measurement. This was necessary because we could not fully extend the fins on several specimens. Measurements taken from live specimens (*L. goodei, L. parva, F. notatus*) show that these are reliable estimates of fin area (Fuller and Brockelsby unpublished data). Caudal fins were visible, allowing us to measure the area directly by tracing the outline of the caudal fin.

We took two approaches to measuring fin shape for dorsal, anal, and caudal fins. First, we measured the ratios of specific rays. For both the dorsal and anal fins, we calculated the mean of the two posterior fin ray lengths and the mean of the two anterior fin ray lengths and calculated their ratio (posterior/anterior ratio). For the caudal fin, we measured the ratio of the median caudal ray to the mean of the dorsal and ventral rays. Second, we estimated the shape using principal components analysis, considering the ray measurements and base length for each fin. For ease of interpretation, we present results for shape as calculated by the ratios. However, analyses of PC scores produce similar patterns (Fuller unpublished results).

### 1.1 Molecular Phylogenetics

We aimed to perform a comparative analysis of fin trait evolution across fundulids. Previous studies have reported the phylogenetic relationships for fundulids, but *Leptolucania ommata* was not included in many of these. Therefore, we utilized molecular sequence data from the most taxon‐complete molecular phylogeny published for fundulids thus far by Ghedotti and Davis (2013), which included sequences for 41 of 43 species of fundulids. Outgroup sequence data included in our analysis are also from Ghedotti and Davis (2013), including representatives of Profundulidae, Goodeidae, Cyprinodontidae, Aphaniidae, Anablepidae, Procatopodidae, Poeciliidae, Valenciidae, Aplocheilidae, and Rivulidae. Non‐molecular data in Ghedotti and Davis (2013) were excluded as these characters create problems for estimating branch lengths. Our dataset includes sequences from two nuclear genes (RAG1 and glyt) and two mitochondrial genes (cytb and COI). Sequences were obtained from GenBank using NCBI e‐utilities (Kans 2013) and aligned using MAFFT v7.490 with default parameters (Katoh and Standley 2013). Sequence concatenation and file conversion were performed using phyx (Brown et al. 2017). We used ModelFinder implemented in IQ‐TREE 2 to identify the best‐fit substitution model and partitioning scheme (Kalyaanamoorthy et al. 2017; Minh et al. 2020).

We performed a time‐calibrated Bayesian analysis in BEAST v2.6.7 (Bouckaert et al. 2019). We constrained the root age based on the divergence of fundulids from Aplocheiloidei of 73.6 Ma (95% credible interval 66.8–79.6 Ma) estimated by Hughes et al. (2018) in a study on ray‐finned fish phylogeny based on genomic data that was calibrated using 31 fossil calibrations. We also constrained the monophyly of the uncontroversial clades Cyprinodontoidei, Alopcheiloidei, and all sampled families (outgroup families and Fundulidae); we did not constrain the monophyly of internal relationships within Fundulidae. Based on the results of ModelFinder, we partitioned the dataset and specified the TIM2 + F + I + G model for mitochondrial sequences and the TNe + I + G model for nuclear sequences. We specified the optimized relaxed clock model and the birth‐death tree prior linked across both partitions. All other priors were retained as default. We ran two independent MCMC runs for 10,000,000 generations, storing every 5000 generations. We assessed convergence by manually evaluating whether effective sample size (ESS) values for parameters were greater than 200 using Tracer v1.7.2 (Rambaut et al. 2018) and discarded the first 10% of samples from each run as burn‐in. From the resulting posterior distribution, we used TreeAnnotator to summarize a Maximum Clade Credibility phylogeny with median heights, which we used for comparative phylogenetic analyses.

### 1.2 Statistical Analysis of Fins

Most traits were positively associated with standard length, which varied across species. Fin areas, fin ray lengths, fin base lengths, and some shape variables were positively correlated with standard length (Table 2). We, therefore, used residuals as a measure of relative size and relative shape. For all traits, we used the residuals from a model that considered the natural log of the trait as a function of the natural log of standard length.

**TABLE 2:** Pearson correlation coefficients between standard length (mm) and fin traits.

Trait	*R*
Dorsal fin area (mm^2^)	0.89
Mean dorsal ray fin length (mm)	0.88
Dorsal fin base length (mm)	0.92
Anal fin area (mm^2^)	0.81
Mean anal fin ray length (mm)	0.93
Anal fin base length (mm)	0.73
Caudal fin area (mm^2^)	0.9
Mean caudal fin ray length(mm)	0.94
Caudal fin peduncle Width (mm)	0.94
Dorsal fin shape (back/front)	0.02
Anal fin shape (back/front)	−0.21
Caudal fin shape (middle/ends)	−0.32

*Note:* *N* = 363 individuals.

Our overarching goal was to determine which fins show evidence of sexual dimorphism in size and shape and whether the levels of sexual dimorphism differed across species. We used general linear models to examine the effects of species, sex, and their interaction on fin traits. We analyzed the significance of these terms using a type 3 model in the ‘car’ package using the ‘options (contrasts = c (“contr.sum”, “contr.poly”))’ statement in R. We then used these models to calculate the effect sizes for sex for each species, which produced a standardized measurement for the difference between males and females relative to the residual error of the model. Specifically, sexual dimorphism was calculated as the difference in means between males and females for each species divided by the pooled standard deviation (i.e., the square root of the model mean square error). We used these estimates of species‐specific sexual dimorphism to calculate the average and 95% confidence limits for the entire family. Estimates of sexual dimorphism and their 95% confidence limits were calculated using the ‘emmeans’ package. We used a sequential bonferonni correction to examine the statistical significance of each term, using the ‘holm’ correction in the p‐adjust function in R.

After determining whether there were differences among species, we next asked whether those differences were attributable to a meaningful phylogenetic signal in the data. For both species means and estimates of sexual dimorphism for each species, we calculated both K and lambda. We did this using a phylogeny obtained from Rabosky et al. (2018), which lacked *Leptolucania ommata*, and our newly derived phylogeny (above). Results were qualitatively identical. K measures the ratio of the variation among clades to the variation within clades (Blomberg et al. 2003; Revell and Harmon 2022). High variance among clades corresponds to a high phylogenetic signal. A significant value for K indicates that the phylogenetic signal in the data is higher than expected, given the data at hand. Lambda is a scaling parameter that calculates whether the similarities observed between species for a given trait result from shared evolutionary histories (Pagel 1999). A significant value for lambda indicates that we can reject the null hypothesis of no phylogenetic signal (i.e., whether it differs from zero). Greater values indicate a stronger phylogenetic signal for a trait, and inversely, smaller values (those that approach zero) indicate a weaker phylogenetic signal.

Finally, to visualize the size and shape of each fin for each combination of species and sex, we calculated the least square means for each ray length and the base length for all three fins. We did this using separate models for each species that considered the effects of sex and the log of standard length on the log trait value. We then back‐calculated the average fin ray length and base length onto the original scale. For each combination of species and sex, we estimated the average length from the origin for each of the fin rays. The first fin ray was located at the origin (0); the first quartile ray was located at one‐fourth of the length of the fin base; the median ray was situated in the middle of the fin base, the third quartile ray was located at three‐quarters of the length of the fin base, and the last ray was located at the end of the fin base. This allowed us to visualize base length, fin ray length, and shape for each combination of species and sex.

All analyses were performed in R (version 4.3.2). Raw data and R scripts can be found at (https://doi.org/10.5061/dryad.wm37pvmz6).

## 2 Results

### 2.1 Phylogeny

**FIGURE 2:**
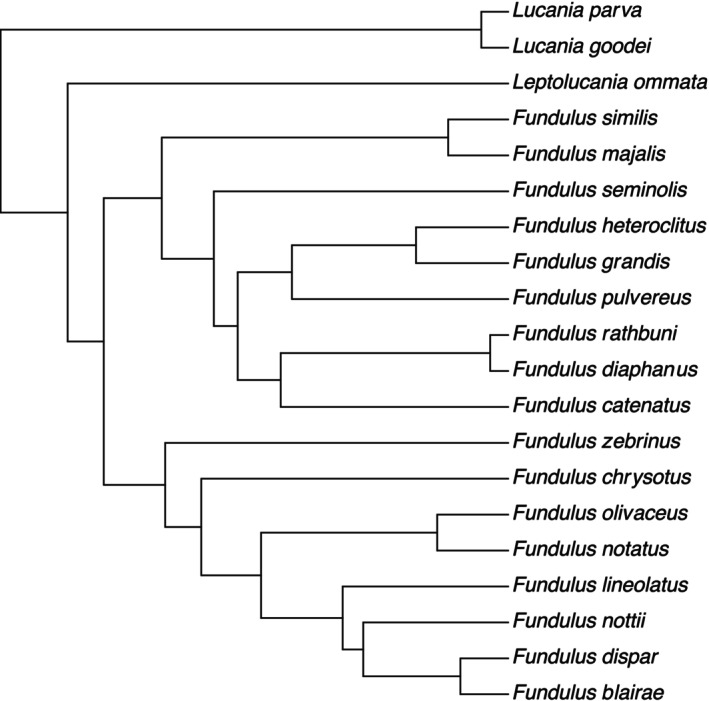
Pruned median phylogenetic tree.

Figure 2 shows the median phylogenetic relationships for the 20 species, including *Leptolucania*. Unsurprisingly, the results are similar to a prior molecular‐only analysis reported by Ghedotti and Davis (2013). The new phylogeny differs in including *Leptolucania*, which was not included in the Ghedotti and Davis (2013) three‐gene analysis since it is only represented by COI. Ghedotti and Davis (2013) had *Leptolucania* in their complete combined analyses (molecular+non‐molecular), where they consistently recovered its placement as sister to *Lucania* with moderate to strong support across analyses. In our complete phylogeny, we recover *Leptolucania* and *Wileyichthys* (0.825 PP). Otherwise, our phylogeny does not present any novel phylogenetic relationships that strongly contradict prior results from phylogenetic and phylogenomic analysis with decent ingroup and outgroup sampling.

### 2.2 Evidence of Sexual Dimorphism

**FIGURE 3:**
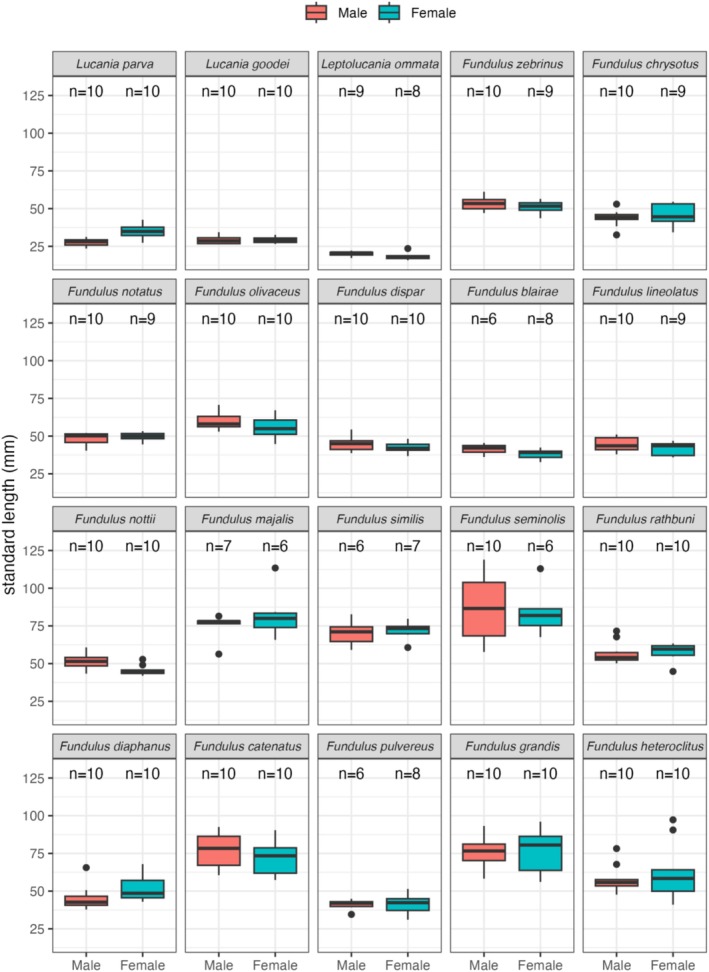
Box plots of male and female standard lengths (mm) for all species. Sample sizes are indicated for each.

There was little evidence for widespread sexual dimorphism across the family for standard length (Table 3, Figure 3). Due to the high correlations between standard length and all fin ray and base lengths (Table 2), we used the residuals from a regression as a size‐corrected measure of the lengths of fin rays and fin bases (see methods). All discussion of fin traits refers to size‐corrected (i.e., residual) traits.

**TABLE 3:** Analyses of standard length, fin area, fin base length, average fin ray length, and fin shape for dorsal, anal, and caudal fins.

Trait	Species *F*	Sex *F*	Species × Sex *F*	Sex dimorph	K (species)	Lambda (species)	K (SD)	Lambda (SD)	PIC R (male–female)
Log (standard length)	**129.43**	0.001	1.92	0.004	**1.33**	**0.99**	0.21	0.28	**0.87**
Resid dorsal area	**24.61**	**692.27**	**2.5**	**2.81**	0.49	0.64	0.33	< 0.00	**0.73**
Resid dorsal base	**41.85**	**206.58**	1.53	1.53	0.41	0.66	0.25	< 0.00	**0.88**
Resid dorsal ray length	**20.08**	**639.84**	**5.26**	**2.7**	0.15	< 0.00	0.6	0.89	**0.87**
Resid dorsal shape	**7.93**	**228.51**	**2.69**	**1.61**	0.41	0.63	0.25	< 0.00	0.3
Resid anal area	**32.13**	**682.22**	**2.23**	**2.79**	0.2	< 0.00	0.16	< 0.00	**0.95**
Resid anal base	**26.89**	**483.68**	**3.51**	**2.35**	0.31	0.44	0.33	< 0.00	**0.87**
Resid anal ray length	**24.08**	**263.62**	**5.59**	**1.73**	0.18	< 0.00	0.38	0.61	**0.88**
Resid anal shape	**13.79**	**298.31**	**3.50**	**1.84**	0.54	**0.76**	0.52	0.69	**0.61**
Resid caudal area	**19.35**	**29.59**	**2.04**	**0.58**	0.21	0.05	0.77	1.01	**0.95**
Resid caudal base	**49.82**	**104.26**	1.64	**1.09**	0.18	< 0.00	0.42	0.51	**0.97**
Resid caudal ray length	**12.97**	**7.11**	1.2	**0.28**	0.25	0.14	0.14	< 0.00	**0.76**
Resid caudal shape	**23.6**	**5.52**	1.04	**0.25**	0.48	**0.65**	0.49	< 0.00	**0.95**

*Note:* df Species F‐tests (19,323). df Sex F‐tests (1323). df Species × Sex F‐tests (1323). With the exception of standard length, all analyses are on the residuals (resid) from a linear regression of log trait value on log standard length. *F*‐values are shown for Species, Sex, and the interaction of Species × Sex. Overall levels of sexual dimorphism are also shown. Metrics of phylogenetic signal (K and lambda) are shown for species means and species values for sexual dimorphism (SD). The phylogenetic independent correlation (PIC) between male and female species means is shown. Values in bold show *p* < 0.05 after a sequential Bonferroni adjustment (13 tests for each statistical term).

**FIGURE 4:**
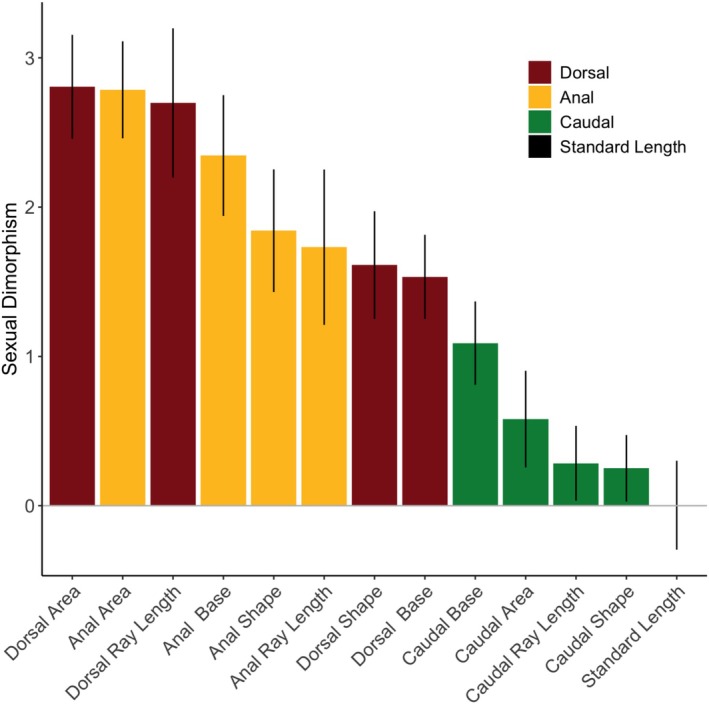
Sexual dimorphism levels averaged across species for standard length, residual area, residual base length, residual average ray length, and residual shape for anal, dorsal, and caudal fins. Bars indicate 95% confidence intervals.

**FIGURE 5:**
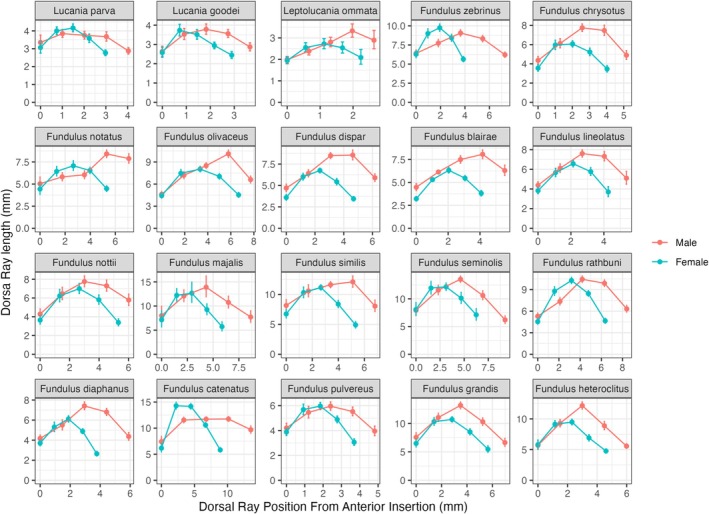
Dorsal ray fin lengths as a function of sex and species. Mean dorsal ray lengths were calculated using least square means from a model of species, sex, their interaction, and the log of standard length on the log of the trait values. Dorsal ray position along the base was estimated using the 1st, 25th, 50th, 75th, and 99th locations based on the average dorsal fin base length for each combination of sex and species. The least‐square means + SE were then back‐calculated onto the original scale.

**FIGURE 6:**
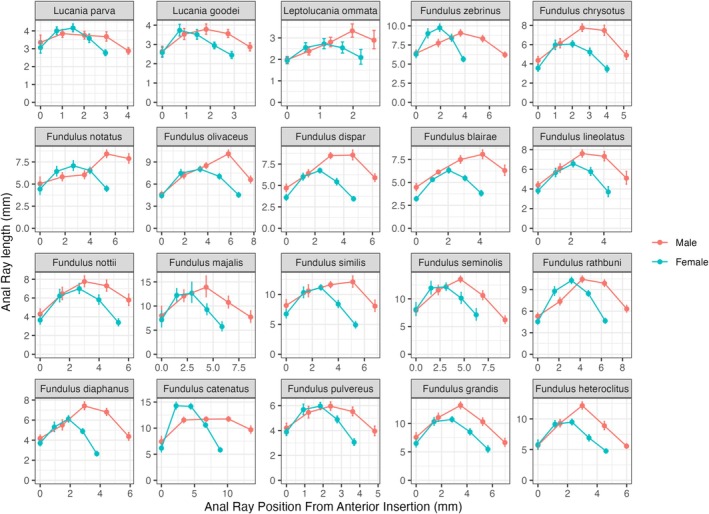
Anal ray fin lengths as a function of sex and species. Mean anal ray lengths were calculated using least square means from a model of species, sex, their interaction, and the log of standard length on the log of the anal fin ray lengths. Anal ray positions along the base were estimated using the 1st, 25th, 50th, 75th, and 99th locations based on the average dorsal fin base length for each combination of sex and species. The least‐square means + SE were then back‐calculated onto the original scale.

Sexual dimorphism was quite high for dorsal and anal fin traits (i.e., area, ray length, and base length). Tables 3 and 4 show highly significant effects of sex for all dorsal and anal fin traits, and Figure 4 shows the average levels of sexual dimorphism for all traits. Sexual dimorphism in dorsal and anal fin area was high (dorsal fins: 2.8, 95% CL 2.5–3.2; anal fins: 2.8, 95% CL 2.5–3.1, Figure 4). For reference, a value of zero means no difference between the two sexes, and an effect size of 0.8 is typically considered large (Cohen, 1988). On average, dorsal fins were approximately 46% larger, and anal fins were 55% larger in males than females. Not surprisingly, both fin ray length and fin base length were also highly sexually dimorphic for both dorsal and anal fins (dorsal‐fin ray length: 2.7, 95% CL 2.2–3.2; anal‐fin ray length: 1.7, 95% CL 1.2–2.5; dorsal‐fin base length: 1.5, 95% CL 1.3–1.8; anal‐fin base length: 2.34, 95% CL 1.9–2.7) (Tables 3 and 4; Figures 5 and 6). On average, dorsal‐fin ray lengths were approximately 26% longer, and anal‐fin ray lengths were 14% greater for males than females. Likewise, dorsal‐fin base lengths were 15% larger, and anal‐fin base lengths were 34% larger in males than females.

**TABLE 4:** Analyses of individual fin ray lengths.

Fin Ray Lengths	Species *F*	Sex *F*	Species × Sex *F*	Sex Dimorph	K (species)	Lambda (species)	K (SD)	Lambda SD	PIC R (male–female)
Resid Dorsal Fin 1st Ray	**14.76**	**103.17**	**3.93**	**1.08**	0.17	0.00007	0.26	0.00007	**0.72**
Resid Dorsal Fin 1st quartile	**11.46**	**129.25**	**3.04**	**1.21**	0.26	0.00006	0.27	0.00007	**0.63**
Resid Dorsal Fin median	**17.32**	**327.56**	**2.87**	**1.93**	0.22	0.00007	0.42	0.00005	**0.86**
Resid Dorsal Fin 3rd quartile	**10.27**	**628.02**	**4.61**	**2.67**	0.15	0.00007	0.45	0.00007	**0.72**
Resid Dorsal Fin Last Ray	**15.85**	**562.82**	**3.92**	**2.53**	0.12	0.00718	0.50	0.58502	**0.88**
Resid Anal Fin 1st Ray	**6.98**	**39.33**	1.19	**0.67**	0.39	0.00007	0.48	0.00008	**0.78**
Resid Anal Fin 1st quartile	**11.08**	3.09	**2.75**	−0.19	0.22	0.00007	0.15	0.00007	**0.67**
Resid Anal Fin median	**22.82**	**59.44**	**8.44**	**0.82**	0.23	0.00006	0.36	0.00007	**0.67**
Resid Anal Fin 3rd quartile	**27.21**	**462.09**	**6.52**	**2.29**	0.23	0.44880	0.37	0.36765	**0.78**
Resid Anal Fin Last Ray	**18.50**	**465.12**	**7.79**	**2.3**	0.21	0.26987	**0.75**	**0.92354**	**0.81**
Resid Caudal Ray Dorsal	**7.90**	3.13	0.99	0.19	0.26	0.00007	0.21	0.00007	**0.75**
Resid Caudal Ray Mid	**30.17**	**15.84**	1.26	**0.42**	0.34	**0.57402**	0.33	0.00006	**0.94**
Resid Caudal Ray Ventral	**5.00**	0.62	1.04	0.08	0.23	0.00007	0.10	0.00007	0.45

*Note:* All analyses are on the residuals (resid) from linear regression of log trait value on log standard length. *F*‐values are shown for Species, Sex, and the interaction of Species × Sex. Overall levels of sexual dimorphism are also shown. Metrics of phylogenetic signal (K and lambda) are shown for species means and species values for sexual dimorphism (SD). The phylogenetic independent correlation (PIC) between male and female species means is shown. Values in bold show *p* < 0.05 after a sequential Bonferroni adjustment (13 tests for each statistical term).

In contrast, levels of sexual dimorphism were lower for caudal‐fin traits (Tables 3 and 4; Figure 4). The average levels of sexual dimorphism were less than 1.1 for all caudal‐fin traits (caudal‐fin area: 0.58, 95% CL 0.26–0.91; caudal‐fin ray length: 0.28, 95% CL 0.03–0.53; caudal‐peduncle width: 1.09, 95% CL 0.81–1.37), whereas levels of sexual dimorphism ranged from 1.5 to 2.8 for all measures of dorsal and anal fin size. The caudal‐fin area was approximately 11% greater in males than in females. Caudal‐fin length and peduncle length (i.e., base length) were 2.4% and 6.4% greater in males than females.

The overall patterns in fin shape mirrored those found for fin size. Sexual dimorphism was high for both dorsal and anal fin shape. For dorsal and anal fins, the shape was measured as the ratios of the posterior ray lengths (average of third quartile and last dorsal ray) relative to the anterior ray lengths (average of the first and first quartile dorsal ray). Males had higher posterior/anterior ratios than females across all species (Table 3, Figure 4). Across all species, the average level of sexual dimorphism was 1.63 (95% CL 1.2–2.0) for dorsal‐fin shape and 1.84 (95% CL 1.43–2.25) for anal‐fin shape.

Figures 5 and 6 show the estimated means of the dorsal and anal ray lengths for each sex and species. The x‐axis shows the position of dorsal rays scaled to the average fin base length for each sex and species. The graphs show clear differences in ray length, fin base, and area. For both the dorsal and anal fins, the levels of sexual dimorphism were higher on the rear portion of the fins. Individual ANOVAs on dorsal and anal fin ray lengths showed higher levels of sexual dimorphism for the third quartile and last ray (Table 4, Sexual Dimorphism > 2) in comparison to the first and first quartile dorsal rays (Sexual Dimorphism ~1).

Sexual dimorphism in caudal‐fin shape was present (Table 3 and Figure 4). Still, it was much lower in magnitude in comparison to anal and dorsal fin shapes (Figure 4). Caudal‐fin shape was measured as the ratio of the middle ray length to the mean of the dorsal and ventral ray lengths. Overall, sexual dimorphism was 0.25 (95% CL 0.028, 0.47), meaning that males had slightly more rounded caudal fins than females. However, for many species, sexual dimorphism did not differ from a null value of zero. Analysis of individual rays shows no sexual dimorphism present in the ventral or dorsal caudal rays but detectable levels of dimorphism in the median caudal ray.

### 2.3 Species Differences in Sexual Dimorphism

Species varied in the levels of sexual dimorphism for the measured traits of all fins. The interaction between Sex and Species was much lower in magnitude than the individual effects of Sex and Species (Tables 3 and 4). Still, the interaction between Sex and Species was statistically significant for all fin traits except for caudal‐fin shape, caudal‐fin length, and dorsal‐fin base length.

**FIGURE 7:**
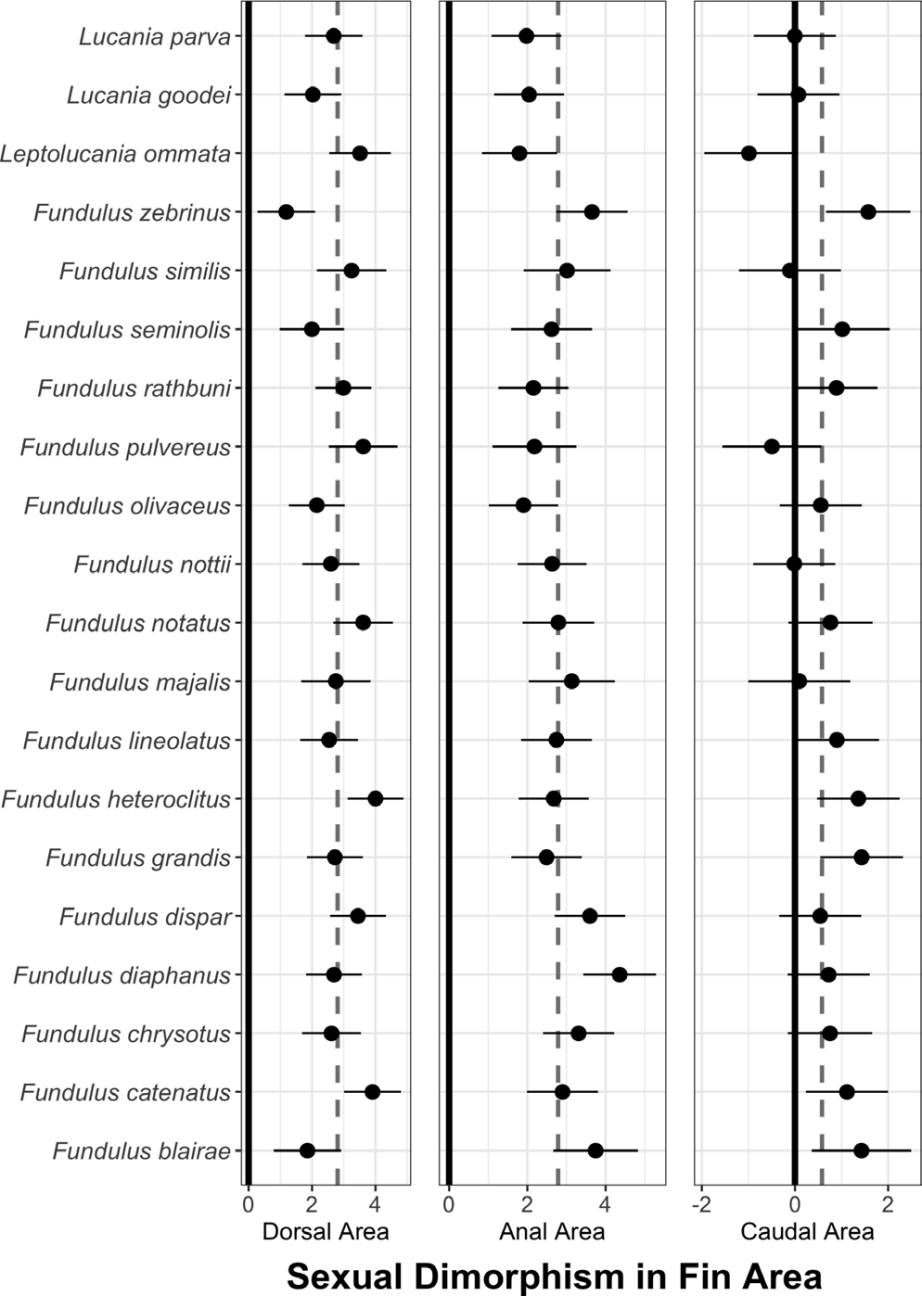
Sexual dimorphism in residual dorsal, anal, and caudal fin areas. Residuals were calculated from a regression of the log of the trait value on log of standard length. The black line at zero shows the null expectation of no sexual dimorphism. The dashed line shows the average level of sexual dimorphism across species. Sexual dimorphism was calculated as the effect size due to sex for each species.

All species had sexual dimorphism in dorsal fin area significantly different from zero. Still, the magnitude of sexual dimorphism varied by more than 2.5x across species (Figure 7). Sexual dimorphism in *F. catenatus* and *F. heteroclitus* was 2.5× greater than in *F. zebrinus* . In most species, there was significant positive sexual dimorphism in both dorsal‐fin base length and dorsal‐fin ray length. In other words, in most species, males had greater dorsal‐fin base length and dorsal‐fin ray length than females. In *F. zebrinus*, males had longer dorsal‐fin bases than females, but females had longer fin ray lengths (Figure 5).

Likewise, all species had sexual dimorphism in anal fin area that differed from zero, but there was over a two‐fold difference in sexual dimorphism across species ranging from 1.90 ( *F. olivaceus* ) to 4.36 ( *F. diaphanus* ) (Figure 7). In most species, males had larger anal‐fin base lengths and anal‐fin ray lengths, but there were a few deviations from this pattern. Males of *F. zebrinus* had longer anal‐fin bases, but females had longer anal‐fin ray lengths (Figure 6). In both *F. seminolis* and *F. catenatus* , males and females had similar anal‐fin ray lengths, but males had greater anal‐fin base lengths than females.

In contrast, sexual dimorphism was lower across all species for caudal‐fin area. Of the 20 species, 13 lacked significant sexual dimorphism in caudal‐fin area. In six species, males had significantly greater caudal fin area than females, and in one species ( *Leptolucania ommata* ), females had greater caudal area than males. For the caudal‐base length, sexual dimorphism ranged from 0.14 ( *L. goodei* ) to 2.29 ( *F. zebrinus* ). Caudal‐fin ray lengths were particularly variable in the direction of sexual dimorphism (i.e., females of some species possessed longer ray lengths than males). The range of values for sexual dimorphism in caudal‐fin ray lengths ranged from −0.85 ( *L. ommata* ) to 1.17 ( *F. majalis* ).

**FIGURE 8:**
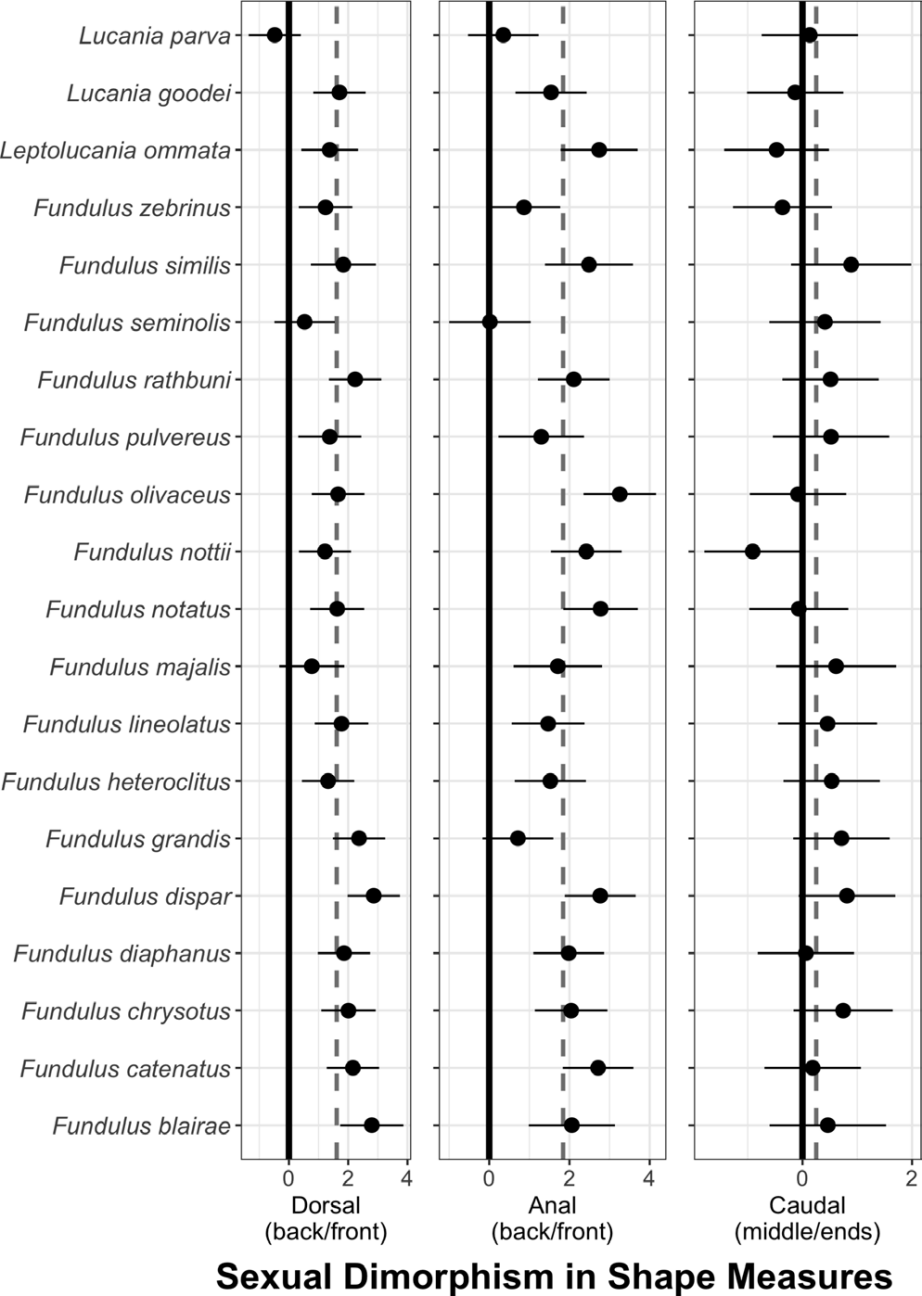
Sexual dimorphism for residual dorsal, anal, and caudal fin shape.

Species differed in levels of sexual dimorphism for dorsal‐fin shape (length of back fin rays/front fin rays), with the lowest levels in *L. parva* (−0.52) and the highest in *F. blairae* (2.91) (Figure 8). However, the shape analyses for most species revealed males with longer posterior rays than females (Figure 5). Similarly, there were species differences in the levels of sexual dimorphism for anal fin shape, with the lowest levels in *F. seminolis* (−0.04) and the highest levels in *F. olivaceus* (3.57). Most species had significant levels of sexual dimorphism in the posterior end of the anal fin, with males typically having greater ray lengths (Figure 6). There was no statistically significant variation across species in sexual dimorphism in caudal fin shape (Table 3).

### 2.4 Correlations Between Sexual Dimorphism

Pearson correlations on phylogenetic independent contrasts indicated that most traits were not correlated in levels of sexual dimorphism. In other words, species with high levels of sexual dimorphism in dorsal‐fin area did not necessarily have high levels of sexual dimorphism in anal‐fin area. The one notable exception is for dorsal and anal fin shape, where a high correlation was present (*R* = 0.658, *p* = 0.0022, Table 5). The correlations between sexual dimorphism in dorsal and caudal fin traits tended to be negative, and one correlation (sexual dimorphism in dorsal and caudal fin ray length, *R* = −0.48, *p =* 0.037) was marginally significant but did not remain so after a correction for multiple tests.

**TABLE 5:** Pearson correlations on phylogenetic independent contrasts between sexual dimorphism in different fin components.

Trait‐Trait	*R*	Unadjusted *P*	Adjusted *P*
Dorsal—Anal Area SD	−0.108	0.65980	1.0000
Dorsal—Anal Base Length SD	0.158	0.51770	1.0000
Dorsal—Anal Ray Length SD	0.203	0.40440	1.0000
Dorsal—Anal Shape SD	**0.658**	**0.00218**	**0.02616**
Dorsal—Caudal Area SD	−0.386	0.10240	1.0000
Dorsal—Caudal Base Length SD	−0.115	0.64020	1.0000
Dorsal—Caudal Ray Length SD	−0.480	0.03732	0.41052
Dorsal—Caudal Shape SD	0.205	0.40026	1.0000
Anal—Caudal Area SD	−0.036	0.88260	1.0000
Anal—Caudal Base Length SD	−0.015	0.95270	1.0000
Anal—Caudal Ray Length SD	0.291	0.22660	1.0000
Anal—Caudal Shape SD	−0.089	0.71719	1.0000

*Note:* DF = 17 in all cases. Unadjusted and adjusted *p*‐values after a sequential Bonferonni correction for 12 tests are shown.

### 2.5 Phylogenetic Signals in Trait Means and Sexual Dimorphism

A strong phylogenetic signal across species was present for standard length (i.e., statistically significant values for K and/or lambda, Tables 3 and 4). Only two other traits (anal‐fin shape and caudal fin shape) showed evidence of a phylogenetic signal across species means. Despite differences in sexual dimorphism among species, there was little evidence for a phylogenetic signal in sexual dimorphism in dorsal and anal fin traits. Sexual dimorphism was marginally significant in anal fin ray length and caudal fin area, but these patterns did not remain statistically significant (Table 3). There was evidence for a phylogenetic signal in sexual dimorphism for caudal fin area (Table 3).

For nearly all traits, the means for males were strongly correlated with the means for females (Pearson Correlation on Phylogenetic Independent Contrasts, Table 5, *p <* 0.006 for all tests). The notable exception was for dorsal‐fin shape (*R =* 0.299, *p* = 0.214).

## 3 Discussion

The goal of this study was to determine which fins show the highest levels of sexual dimorphism and whether these patterns differed among species. We also asked whether there are phylogenetic signals indicating that closely related species share similar levels of sexual dimorphism for individual traits and searched for correlations in the levels of sexual dimorphism between fins. We found very high levels of sexual dimorphism in anal and dorsal fin size and shape and lower levels of sexual dimorphism in caudal fin size and shape. We also found that species differed in the magnitude of sexual dimorphism. There was a strong phylogenetic signal for standard length but little evidence for a phylogenetic signal in most fin traits, after controlling for standard length. Similarly, there was little evidence that sexual dimorphism was correlated across different fins, with the notable exception of anal and dorsal fin shapes. Species with high levels of sexual dimorphism in dorsal‐fin shape also had high levels of sexual dimorphism in anal‐fin shape. We discuss the implications of these findings below.

There was striking sexual dimorphism in anal and dorsal fin size and shape across all 20 of the fundulids that we measured in this study. Furthermore, the dimorphism in anal and dorsal fin size is attributable to changes in fin ray lengths (particularly in the posterior fin rays) and the base length of these fins. The strength of this pattern across a diversity of species spanning magnitudes of order in body size suggests the presence of shared sexual selection for larger fins in males compared to females. Within fundulids, this pattern has been documented in one species (Welsh et al. 2013; Welsh and Fuller 2015). Older PhD theses have commented verbally that males appear to have larger dorsal and anal fins than females (Foster 1967). Beyond fundulids, other studies have found evidence for sexual dimorphism in dorsal and anal fin size in teleosts, including gar, minnows, blennies, cichlids, salmonids, and medakas (e.g., Chervinski 1965; McCart 1965; Ostrand et al. 2001; McGrath and Hilton 2012; Englmaier et al. 2022). Still, the overwhelming pattern and its ubiquity across fundulids is a genuine contribution to our understanding of this group.

The striking presence of sexual dimorphism in dorsal and anal fins across a diversity of fundulid species spanning magnitudes of order in body size suggests the presence of shared sexual selection for larger dorsal and anal fins in males compared to females. Dorsal and anal fins can serve multiple functions, including swimming/maneuvering in the water column, male display towards females and rival males, the movement of sperm during fertilization, and grasping of females during spawning. In medaka ( *Oryzias latipes* ), female preference for males with longer dorsal and anal fins has been shown (Fujimoto et al. 2014). Males of many species use their dorsal and anal fins to clasp female mates during spawning (Newman 1907). The removal of anal fins has been shown to reduce fertilization success (Koseki et al. 2000). During territorial fights, males of some species will flare their fins to establish dominance (Thompson and Sturm 1965; Fuller 2001; McGhee et al. 2007; Goldberg et al. 2019). In Mediterranean killifish, success in male–male dominance interactions has been attributed to greater dorsal‐fin height (Malavasi et al. 2010). In short, dorsal and anal fins serve multiple functions. Teasing these apart will require careful experimentation that alters fin size and/or shape and examines the functional consequences on male courtship, male competition, and fertilization success. Alternatively, phylogenetic approaches may provide insight into the evolution of sexual dimorphism and large fin size in males if taxa vary in the functions of anal and dorsal fins.

This study also found modest evidence for sexual dimorphism in caudal fin size and shape. On average, across the 20 fundulid species measured, the magnitude of sexual dimorphism in caudal fin size was greater than zero, although the levels of sexual dimorphism in many individual species did not differ significantly from zero. This finding of low levels of sexual dimorphism in caudal fin traits is consistent with an in‐depth study of fin dimorphism in two species of *Lucania* (Brockelsby, Davis, Fuller, unpublished result). While this sexual dimorphism is small in magnitude compared to anal and dorsal fins, it is present.

Across teleosts, sexual dimorphism in caudal fin traits does not appear commonly in the literature, with the notable exception of swordtails, where female preference is biased towards larger male caudal fins (i.e., longer swords; Rosenthal and Evans 1998). Sexual size dimorphism has been reported in caudal fin size and shape in other species, including guppies (Karino and Matsunaga 2002) and spotfin shiners (Pyron et al. 2007), with males having larger caudal fins. The extent to which the caudal fin is involved in dominance or courtship displays in fundulids is unknown. It is currently unclear whether the low levels of sexual dimorphism in fundulid caudal fins represent the result of weak sexual selection or a correlated response for increased fin sizes in dorsal and anal fins. Here, the median caudal fin ray was, on average, longer in males than in females. Presumably, this would increase drag, making sustained swimming more difficult (Blake 2004; Langerhans 2008).

While the direction of sexual dimorphism in fundulid dorsal and anal fins was strikingly consistent, the magnitude of the dimorphism was also variable. For example, sexual dimorphism in anal‐fin bases varied nearly four‐fold among species. We note that our sample sizes were somewhat low in this study. Most combinations of species and sex had 10 individuals, but a few species within our dataset contained only six individuals for a given sex. However, we took other statistical approaches to measuring sexual dimorphism and obtained similar results. Multiple forms of selection (i.e., female preference, male competition, fertilization) have been correlated with sexually dimorphic fin traits. Variation in the strength of these selection pressures may contribute to the species variation observed in this study. These species differ in the duration of the breeding season, possibly due to latitudinal temperature differences; other species spawn on a lunar cycle. Likewise, variation in the costs to ornamentation due to differences in predation and/or locomotor costs, possibly due to stream speed, may affect these patterns.

Surprisingly, we found little evidence for a phylogenetic signal in sexual dimorphism for most traits. Many other studies have shown strong phylogenetic signals in male ornamentation/secondary sex traits (Kang et al. 2013; Ciccotto and Mendelson 2016; Goldberg et al. 2019). In this study, we found a strong phylogenetic signal for standard length but not for sexual dimorphism in standard length. We did find moderate evidence for a phylogenetic signal in the sexual dimorphism for the caudal‐fin area. *Lucania* showed the lowest levels of sexual dimorphism, while members of the *Fundulus* genus had significantly higher levels. The lack of a phylogenetic signal for most sexually dimorphic fin traits suggests that the magnitude of selection varies across populations and species. Variation in water flow can influence fin shape (Langerhans 2008; Binning and Roche 2015). Large ornamental fins can negatively affect swimming performance, and habitats with lower predation risk might allow for greater sexual dimorphism in fin size (Sowersby et al. 2022). Selection pressures may act on each species differently for many of the fin traits observed, resulting in differences in the levels of sexual dimorphism despite evolutionary relatedness.

Our study also searched for correlations across different fins, which could indicate shared developmental pathways for fin growth and/or similar forms of selection. We found correlations between sexual dimorphism in the posterior/anterior shape in dorsal and anal fins, but whether these shape traits share a common developmental pathway will require further investigation. Nonetheless, the observed correlation in sexual dimorphism in fin shapes may indicate that similar mechanisms regulate growth in these regions. Males and females do not differ in fin traits when fins first emerge in development (Fuller unpublished). Instead, sexual dimorphism in fin size and shape is thought to develop over time, with some species showing a rapid period of secondary growth as fish approach sexual maturity. Studies in medaka have tracked fin growth over time and shown distinct periods of sex‐specific growth, with males experiencing accelerated growth compared to females (Kawajiri et al. 2015; Im et al. 2016). Males and females likely face similar selection pressures during early life stages, as juveniles use their fins solely for locomotion. Differentiation in fin morphology at the time of sexual maturity might enable the secondary functions that male fins employ during adult life stages. The pronounced variation among species in sexual dimorphism in fundulids sets a ripe stage for investigations into the extent to which there is variation in the (a) onset of sex‐specific growth trajectories, (b) growth in particular rays (i.e., posterior versus anterior), and (c) the social and environmental cues that trigger the development of sexual dimorphism.

In conclusion, this study found a strikingly consistent pattern in sexual dimorphism in anal and dorsal fins in North American killifish (Fundulidae). Males possessed larger dorsal and anal fins with longer fin rays and base lengths. Anal and dorsal fin shapes were also sexually dimorphic, with males having larger posterior dorsal fin rays than females. Correlations between levels of sexual dimorphism were observed between the shape of the dorsal and anal fins, which may suggest that these fins have a shared developmental pathway. Most traits lacked a strong phylogenetic signal. There was also striking variation among species in sexual dimorphism in dorsal and anal fin traits. In most species, increased fin area in males was due to a combination of longer fin rays and longer fin base lengths, but a few species did not follow this pattern. In contrast, caudal‐fin size was much less dimorphic between the sexes. Detailed studies of fin development and function will undoubtedly provide greater insight into this pattern. Combined with the results of previous studies, our results show that sexual dimorphism in dorsal and anal fin properties is likely widespread.

## Author Contributions

**Elijah J. Davis:** data curation (equal), formal analysis (equal), funding acquisition (equal), methodology (equal), project administration (equal), writing – original draft (equal), writing – review and editing (supporting). **Kasey Brockelsby:** data curation (supporting), investigation (supporting), methodology (supporting). **Milton Tan:** formal analysis (equal), investigation (equal), writing – original draft (supporting), writing – review and editing (supporting). **Rebecca C. Fuller:** conceptualization (lead), formal analysis (equal), funding acquisition (equal), methodology (equal), project administration (equal), writing – original draft (equal), writing – review and editing (lead).

## Conflicts of Interest

The authors declare no conflicts of interest.

## Data Availability

Here is a link to dryad where the reviewers can see the data and R scripts: https://doi.org/10.5061/dryad.wm37pvmz6.
